# Systematic Quality Improvement in Medicine: Everyone Can Do It

**DOI:** 10.5041/RMMJ.10055

**Published:** 2011-07-31

**Authors:** Mark L. Zeidel

**Affiliations:** Department of Medicine, Harvard Medical School, Beth Israel Deaconess Medical Center, 330 Brookline Avenue, Boston, Massachusetts 02138, USA

**Keywords:** Quality improvement, education, training

## Abstract

In this brief review, written from the perspective of a physician-leader who has fostered the development of comprehensive quality improvement efforts at two academic medical centers, I review the need for improvement, some conceptual barriers that must be overcome, the goals of a comprehensive quality improvement (QI) effort, some of the results we have obtained, and some observations on how to develop a culture of continuous improvement in an academic medical center. The mandate for quality improvement is clear; current healthcare is wasteful and error-prone, leading to excessive morbidity and mortality and unsustainably high costs. Successful quality improvement requires the abandonment of two paradigms: the craft model of medical practice and the notion that many forms of harm to patients are not preventable. I will describe how dramatic improvement has been achieved in reducing, by up to 10-fold, rates of central line infections, ventilator-associated pneumonias, peritonitis in peritoneal dialysis patients, and mortality due to cardiac arrest in hospital. I will describe as well how these methods can improve access to out-patient clinics dramatically and enhance the reliability and safety of hand-offs between covering physicians. To develop and maintain systematic quality improvement in all phases of medical care we must articulate a culture in which: everyone working at the medical center makes improvements every day; front-line staff, who know best how the work is done, are empowered to improve the processes of care; and multidisciplinary teams create the protocols that reduce variation that is due to physician preference, leaving only the variation required by the individual needs of patients. I will review as well the crucial elements of education of trainees and faculty members needed to guide and sustain a culture of quality. Finally, I will add some observations on how oversight boards and medical center leaders can help create systematic quality improvement in their medical centers.

## THE MANDATE FOR IMPROVEMENT

Healthcare around the world is expensive, unreliable, and dangerous. In 1999, U.S. experts estimated that medical errors cause 44,000–99,000 preventable deaths per year and account for over $10 billion per year in excessive costs.^1^ Although mortality figures may be accurate, the cost figures are likely to under-estimate vastly the true costs of unreliable, highly variable, and poorly co-ordinated care. What is most tragic is that our technical capabilities have never been better, but we fail repeatedly to use these capabilities to benefit our patients.^2^,^3^ Two major paradigms rampant in current medical practice block quality improvement.

## ABANDONING TWO PARADIGMS

*Paradigm #1: the craft model of care.* Most physicians began medical school with the view that they were becoming craftsmen, who would use their unique skills, mastered over years of schooling and personal training (the “apprenticeship” of internship, residency, and fellowship), to *handcraft* specific diagnostic and treatment regimens that would be optimal for the care of each patient. The craft model promises that this approach will provide the best outcome possible for all patients. As we have noted above, the craft model, which has dominated medical practice for all time, leads to high rates of errors, unacceptably poor outcomes, and massive waste. Can we use industrial design, which standardizes processes, to improve care? The first person to introduce standardization to manufacturing was Eli Whitney, who used interchangeable parts to convert the production of muskets from handcrafting to standard manufacturing.^4^ Unlike handcrafted muskets, which often misfired and which were expensive and time-consuming to produce, muskets made of interchangeable parts were reliable and could be made cheaply and quickly. Within a few years the gunsmith was replaced with the gun factory.

Industrial design has gone through many phases, starting with so-called “scientific management”, which featured processes carefully designed by engineers, and workers who were supposed to do what the managers told them to do.^5^

**Figure 1:**
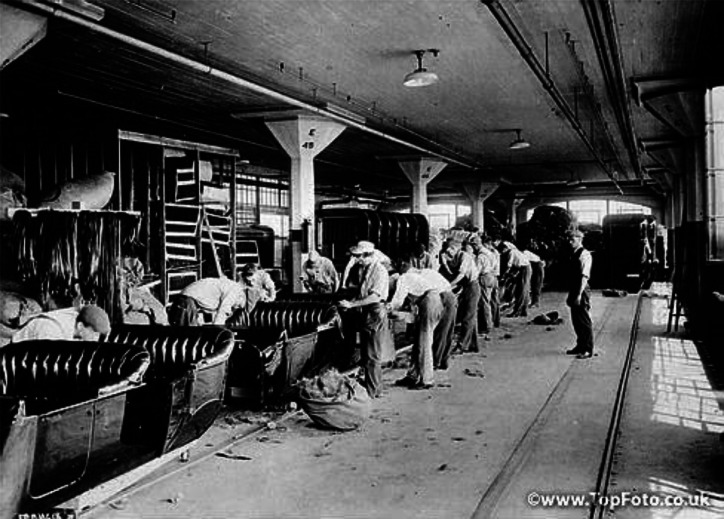
A Ford assembly line, circa 1910. Many physicians associate standardization of care with regimentation akin to the control the foreman in this picture exerts over the workers.

As an aside, when physicians oppose standardization, citing the need for “physician autonomy”, they often believe that the application of protocols will put them in the position of factory workers under the close control of a foreman (Figure 1).The next phase of industrial design involved statistical process control, sampling, and quality control. These approaches were used with great success by Deming and others to enhance American war production in World War II.^6^ Interestingly, the Japanese recognized that they lost the war due in large part to superior U.S. war production. They enlisted Deming in an effort to improve their production techniques; the result was the Japanese manufacturing revolution that made names like Toyota, Mitsubishi, NEC, and others synonymous with quality and innovation. Toyota advanced quality improvement to the system of “lean” or “pull through” production, which lowered costs and improved the quality of its cars dramatically.^7^*Systematic quality improvement seeks to apply the very best elements of industrial design to those elements of health care which are performed again and again in patient care.*

*Paradigm #2: many forms of error are inevitable.* In the movie “It’s a Mad, Mad, Mad, Mad World”, Ethel Merman, who plays an extremely difficult mother-in-law, responds to the statement “These things happen” from her benighted son-in-law (played by Milton Berle) with a battle cry for the quality movement: “These things happen! What kind of an attitude is that? These things happen, because every time these things happen, someone says, ‘These things happen’, and that’s why they happen!”How does this relate to health care? Let me paraphrase Ethel Merman’s character: “Central line infections happen, because every time central line infections happen, someone says, ‘Central line infections happen’, and that’s why they happen!”Of course you can substitute in ventilator-associated pneumonia, peritonitis in peritoneal dialysis patients, cardiopulmonary arrests in hospitals, huge delays in scheduling ambulatory care, and so on. As another example of the “These things happen” mentality, in the U.S., when a frail elderly patient develops delirium in hospital, we call it “sun downing”, as if the delirium is as inevitable as the sunset.^8^*We must disabuse ourselves of the “these things happen” mentality, or we will never improve the quality of care.*

## EXAMPLES OF QUALITY IMPROVEMENT

Approaches to systematic quality improvement make sense if and only if they deliver results. I will describe briefly multiple quality improvement efforts, completed successfully at two different U.S. academic medical centers, as a way of proving the benefits of this approach. These brief vignettes represent a fraction of the total successful quality improvement efforts going on at University of Pittsburgh Medical Center (UPMC) and Beth Israel Deaconess Medical Center (BIDMC).

## CENTRAL LINE INFECTIONS

Centrally placed catheters present major risks of blood-stream infection for patients; they are excellent portals of entry for infection, and patients who develop these infections have high mortality as well as increased morbidity and length of stay. At both UPMC and BIDMC, base-line rates of central line infection were at or just below national norms. At each center we used several interventions: 1) Catheters could only be placed by those who were trained, certified, and had performed enough procedures to maintain competence. Often, simulation was used in initial training. 2) All catheters were placed using five barrier precautions, using standardized line kits. 3) Time outs and documentation were standardized. 4) Vigorous efforts by the leadership and clinical thought-leaders assured that these measures were performed every time, with every line. 5) Because we believed that catheters placed outside of our hospitals were not up to our standards, all such catheters were removed within hours of the patient’s arrival.

**Figure 2:**
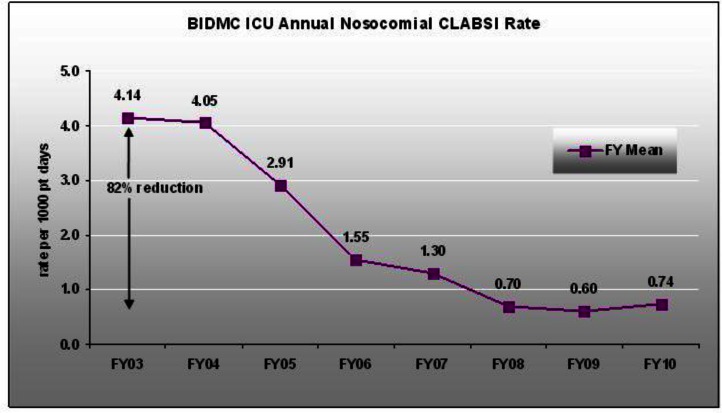
Central line infection rate in ICUs (CLABSI) by quarter, plotted on the ordinate as infections per thousand ICU patient days. FY refers to fiscal year.

As figure 2 shows, at BIDMC these measures reduced central line infection 9-fold, and the improvements have been maintained for years since the interventions began. UPMC and other hospitals in Pittsburgh participated in a joint effort to eliminate these infections; the results were reported in *Morbidity and Mortality Weekly Reports* under the title “Elimination of central line infections: Pittsburgh”. More recently, a collaborative effort across the state of Michigan has resulted in sharp reductions in central line infections state-wide.^9^,^10^

## VENTILATOR-ASSOCIATED PNEUMONIA

Endotracheal tubes provide ready access for mouth flora to enter the lung, often resulting in pneumonia. A large body of evidence has shown that adoption of several measures, including elevation of the head of the bed to 30 degrees, daily awakening of sedated ventilator patients, and frequent assessment of ability to remove the endotracheal tube, reduces the rate of ventilator-associated pneumonia (VAP).^11^ At both UPMC and BIDMC the ventilator bundle was adopted and performed on every patient, every day. The rate of VAP fell 10-fold with adoption of the bundle and has remained at this new lower level for the past 2 years. *With the adoption of these and other quality improvement measures, length of stay in the ICUs fell 20%, permitting the same number of ICU beds to care for 20% more patients. This obviated the need to build an additional ICU to care for increasing ICU volume. Moreover, mortality in the ICU population fell 12%, so that for every 40 patients cared for in the ICUs, one less patient died. Since we care for 6,000 ICU patients per year, this means we now avoid deaths in some 150 patients per year.*

## REDUCING IN-HOSPITAL CARDIOPULMONARY ARRESTS

Retrospective forensic chart studies of patients who have undergone cardiopulmonary arrests reveal that up to 80% of cardiopulmonary arrests are preceded by some indication of physiologic instability, ranging from high fever to high or low pulse, to high or low respiratory rate, to loss of mental status, to marked nursing concern (reviewed in 12,13). At UPMC rapid response teams were developed, which include an intensivist and respiratory and nursing support. At BIDMC the rapid response team consists of the patient’s intern and resident as well as the front-line nurse and a senior nurse. When a patient on a general medical or surgical floor exhibits vital signs or a change in status (including loss of sensorium or a change in condition that causes marked nursing concern) which presage a potential cardiopulmonary arrest, a “trigger” is called. This mandates immediate evaluation by the intern and primary nurse, assisted by the resident and a senior nurse. The physicians must contact the attending physician to go over the patient’s status and the plan for immediate evaluation and management. The trigger is documented using standard forms. All triggers are evaluated by the quality improvement team. In addition, all cardiopulmonary arrests are evaluated, with a forensic examination of the chart, to determine whether a trigger should have been called prior to the arrest. Before we began the intervention, the BIDMC rate of deaths per 1,000 patient days among non-DNR (do not resuscitate), non-intensive care unit patients was 0.95. Since we have implemented the program, this rate has fallen to 0.1–0.2 deaths per 1,000 patient days, and the rate has remained steady for fiscal years 2008, 2009, and 2010. Comparable base-line death rates for hospitals implementing rapid response teams have been 1.1 deaths per 1,000 patient days.^12^,^13^ Although these early rescue approaches appear to have had an important impact on mortality at BIDMC, it has been difficult to demonstrate a similar benefit of rapid response teams in other settings.^12^,^13^ In part this results from the difficulty in conducting randomized controlled studies of quality improvement interventions. In addition, different hospitals have used different approaches to rescuing patients at risk. For example, in many hospitals, detection of a patient at risk brings an intensivist to the bedside. Compelling an intensivist to respond, rather than the patient’s own physicians, may set a psychological barrier that is high enough to inhibit rescue calls that need to be made.

## REDUCING PERITONITIS IN CHRONIC PERITONEAL DIALYSIS PATIENTS

Over a period of two decades, the chronic peritoneal dialysis program at the University of Pittsburgh Medical Center, along with other programs, developed systematic approaches to the prevention of peritonitis among their patients. These included standardized protocols for line care, for performing and teaching the perform-ance of solution changes, and the use of topical antibiotics. Between the early 1980s and the present, the program cut the rate of *Staphylococcus aureus* peritonitis from 0.2 per dialysis year at risk to 0.01–0.02, and it reduced the rate of catheter infections from 0.4–0.5 to 0.05. It is important to note here that, since the patients themselves perform the solution exchanges and care for their catheters, success in reducing peritonitis *arises from improvements in training patients to perform these tasks.* In this case, standardization and improvement in protocols applies to what the patients themselves perform.^14^

## REDUCING WAIT TIMES FOR AMBULATORY CARE

When patients call hospital-based clinics to schedule appointments, they often experience poor telephone service (calls answered by machines, schedulers who do not display appropriate customer service or registration skills), as well as long delays between the day the call is made and the appointment is offered. To improve the telephone experience and timeliness of appointments, we instituted mystery shopping, in which nurses, posing as patients, call each clinic twice monthly and record verbatim the telephone interaction with the schedulers. All results are shared at a monthly meeting of physician and clinic directors and their administrative support leaders. Over a 2-year period customer service and registration skills improved dramatically, and they remain at high levels. The average time between the phone call and the appointment offered fell from 17 to 3 days.

## ASSURING THAT HAND-OFFS BETWEEN HOUSE OFFICERS ARE RELIABLE

In the United States, the on-going restriction of work hours for residents has increased strikingly the number of times that covering physicians sign out patients to one another.^15^ The increased frequency of sign-outs, or hand-offs, increases the risk that vital information will not be transmitted and that resulting mistakes will lead to patient harm. A team of residents at our institution has standardized the process of hand-offs, so as to assure that important information is reliably conveyed from one physician to another. Early results indicate that house officers are far more satisfied with the new system than the prior state, and that errors –both those that do not reach the patient, and those that do – are sharply reduced by the standardized hand-off approach. *It is important to note here that the residents developed this system as part of their quality improvement training, under the guidance of a few faculty advisors. This new system follows the modern industrial design principle of having those who do the work improve the work.*

## GOALS IN CREATING A CULTURE OF QUALITY

At BIDMC we are developing a culture in which the people who are doing the work of healthcare identify and call out problems, and use systematic approaches to fix them, including root cause analysis and standardization of processes of care. importantly, our people increasingly identify quality improvement as an essential component of the care they deliver every day. What are we doing to achieve this culture of quality?

1. *Make quality improvement an explicit component of the mission, communicated constantly by all leaders.* At both BIDMC and UPMC the Board of Directors receives constant reports on quality of care, both through quality improvement committees and in direct reports at full board meetings. Board members become highly educated in the measurement and improvement of quality, and their keen interest in this area is communicated to all staff. The hospital CEO and department chairs communicate the importance of quality improvement constantly to staff and their departments. In the BIDMC Department of Medicine, every faculty meeting, no matter what its main topic, begins with a slide discussing our intense desire to provide to each patient under every circumstance the kind of care we would each want our family members to receive. The chair sends to all faculty members and staff in the Department a weekly newsletter which includes the conference schedule, all publications from the past week, announcements of faculty achievements, and a message from the chair. Most weeks, this message focuses on quality improvement, outlining goals, describing specific projects, and identifying obstacles to success.
2. *Develop and maintain a quality improvement structure which encourages and supports front-line staff in their quality improvement efforts.* The hospital maintains a vigorous quality improvement office, the Silverman Institute for Healthcare Quality, which supports quality improvement efforts in all arenas of care. Each department has its own quality improvement officer. The council of QI officers goes over issues that span departments (as nearly all QI issues do) and evaluates cases in which care did not meet the level of excellence that we expect to achieve. Such cases are reviewed by the chiefs and a board committee if they are particularly difficult or if there are important lessons to be learned across the organization from them. The hospital reports publicly on its progress in achieving quality improvement goals as a means of encouraging all who work at BIDMC to continue to push for enhanced quality of care. The Department of Medicine has a Vice Chair for Quality Improvement; he has clerical, statistical, and epidemiological support. Each clinical division has a chief quality officer. Working with the vice chair and the faculty members in their divisions, the quality officers identify annual quality goals and targets specific for each clinical area. Divisions select quality goals based on the numbers of patients affected, the risk of failure to improve, and the ability to improve care and detect improvement when it occurs. Over the course of the year, the division chiefs, the chair, and the vice chair monitor the progress of each divisional quality improvement effort, to assure that each division achieves its goals.
3. *Invest in training students, residents, fellows, and all staff in quality improvement.* Regret-tably, the concepts of industrial design and quality improvement are not currently taught in most medical schools. Developing and maintaining a culture of quality requires constant training of professional staff. In addition, we have developed strong training programs for residents in quality improvement. Since many of our residents go on to become faculty members, training them in quality improvement reinforces a culture of quality amongst our faculty members. Also, because we have an obligation to provide the most comprehensive and effective clinical training possible, we must assure that they receive excellent training in quality improvement.

I have described briefly efforts to develop a culture of quality at two U.S. academic medical centers. Doing this requires that we abandon the craft model of medical care, and standardize practice, and that we actively discourage the “These things happen” mentality. Creating a structure that enhances quality improvement, from direct Board of Directors involvement to the direct involvement of clinical leaders, to the provision of support to front line-staff, all encourage each caregiver to make quality improvement a part of their daily work. Training students, residents, and staff in quality improvement markedly reinforces the culture. These efforts lead to marked improvements in quality, sharp reductions in the rates of complications, and prevention of unnecessary deaths. Systematic quality improvement is now a professional obligation for all physicians; we all can, and must, do it.

## Abbreviations:

BIDMC: Beth Israel Deaconess Medical Center;
DNR: do not resuscitate
QI: quality improvement
UPMC: University of Pittsburgh Medical Center;
VAP: ventilator-associated pneumonia.

## References

[1] Kohn LT Corrigan JM Donaldson MSeds To Err Is Human: Building a Safer Healthcare System Washington, D.C Institute of Medicine, National Academy Press 2000 25077248

[2] Swensen SJ, Meyer GS, Nelson EC (2010). Cottage industry to postindustrial care – the revolution in healthcare delivery. N Engl J Med.

[3] Zeidel ML, James BC (2002). Improving the quality of healthcare in America: what medical schools, leading medical journals, and federal funding agencies can do. Am J Med.

[4] Green CMcL (1972). Eli Whitney and the Birth of American Technology. Library of America Biography Series.

[5] Taylor FW (1911). The Principles of Scientific Management.

[6] Deming WE (2000). The New Economics for Industry, Government, Education.

[7] Womack JP, Jones DT, Roos D (1990). The Machine That Changed the World..

[8] Shem S (1978). The House of God.

[9] Centers for Disease Control and Prevention (CDC) (2005). Reduction in central line-associated bloodstream infections among patients in intensive care units –Pennsylvania, April 2001 – March 2005. MMWR Morb Mortal Wkly Rep.

[10] Provonost P, Needham D, Berenholtz S (2006). An intervention to decrease catheter-related bloodstream infections in the ICU. N Engl J Med.

[11] Valencia M, Torres A (2009). Ventilator-associated pneumonia. Curr Opin Crit Care.

[12] Sarani B, Scott S (2010). Rapid response systems: from implementation to evidence base. Jt Comm J Qual Patient Saf.

[13] Litvak E, Provonost PJ (2010). Rethinking rapid response teams. JAMA.

[14] Piraino B, Bernardini J, Bender FH (2008). An analysis of methods to prevent peritoneal dialysis catheter infections. Perit Dial Int.

[15] Van Eaton EG, McDonough K, Lober WB, Johnson EA, Pellegrini CA, Horvath KD (2010). Safety of using a computerized rounding and sign-out system to reduce resident duty hours. Acad Med.

